# Analysis and Modeling of the Micro-Cutting Process of Ti-6Al-4V Titanium Alloy with Single Abrasive Grain

**DOI:** 10.3390/ma13245835

**Published:** 2020-12-21

**Authors:** Łukasz Rypina, Dariusz Lipiński, Błażej Bałasz, Wojciech Kacalak, Tomasz Szatkiewicz

**Affiliations:** Faculty of Mechanical Engineering, Koszalin University of Technology, Racławicka 15-17, 75-620 Koszalin, Poland; lukasz.rypina@tu.koszalin.pl (Ł.R.); blazej.balasz@tu.koszalin.pl (B.B.); wojciech.kacalak@tu.koszalin.pl (W.K.); tomasz.szatkiewicz@tu.koszalin.pl (T.S.)

**Keywords:** modeling, micro-cutting, finite element method, grinding, process, chip, blade, pile-ups

## Abstract

Modeling of material displacements in the microcutting zone is complex due to the number and interdependence of factors affecting the results of the process. An important problem in the modeling process is the selection of the constitutive model and its parameters, which will correctly describe the properties of the material under the conditions of triaxial compression, which is characteristic for the areas of the contact zone of the blade and the processed material in abrasive machining processes. The aim of the work was to develop computer models (with the use of the finite element method) of the microcutting process with a single abrasive grain, which were verified with the results of experimental tests. The paper presents the methodology of modeling the processes of microcutting with abrasive grains, whose geometrical models were created based on optical scanning methods. Observations of the microcutting process were carried out with the use of a high-speed camera and an optical profilometer. This enabled a detailed observation of the chip formation process, as well as the analysis of the surface topography of microcutting traces. The results presented in the paper indicate the convergence of the results of the numerical and experimental simulations with regard to the geometric parameters describing the scratches formed in the microcutting process and the compliance of the chip-forming process. Thus, the correctness of the selection of the constitutive model (Johnson Cook equation) and its parameters was demonstrated, as well as the correctness of the applied methodology for creating a geometric model that allowed for a reflection of the geometrical parameters of the abrasive grains that coincided with the real objects, thanks to which it was possible to reflect in detail the phenomena occurring in the vicinity of the abrasive grain tip.

## 1. Introduction

In precise abrasive machining, its result is determined by the phenomena occurring in the vicinity of an abrasive grain. In precision machining, the material separation processes are complex due to the structure and properties of abrasive tools [1], varied geometry of abrasive grain vertexes [2], abrasive grains size, high cutting speeds, locally variable specific cutting resistance and physical properties of the processed material.

The recognition of all the phenomena occurring in the vicinity of abrasive grain vertexes [3], despite advanced measuring techniques, is difficult and in some cases impossible. In order to explain the mechanisms of material removal, research is carried out on the processes of cutting by a single abrasive grain [3,4] by analyzing three phases: rubbing, ploughing and cutting. During the grain operation in the rubbing phase, the material deforms mainly elastically and only slightly plastically. During the penetration of the grain into the material in the plowing phase, grooves and side pile-ups are visible in the processed material. Only in the third cutting phase, with a further increase in the depth of the cut, is there a removal of the processed material in the form of a chip.

Even earlier research works were based on the assumption that the process of a single abrasive grain could be considered in a manner analogous to the orthogonal cutting model developed by Merchant [5,6], which occurs during turning and milling processes. In orthogonal turning, chip formation occurs as the material moves along the wear plane. The separated chip material moves in an almost entirely parallel way to the surface of the blade, whose rake angle is positive, and almost all of the energy is consumed upon chip formation [7]. In order to properly approach the conditions of the grinding process, cutting tests were carried out with blades with negative rake angles [8]. They confirmed the predictions that with the increase of the negative rake angle of the cutting edge, the cutting forces increase and the specific energy of the process increases.

In order to describe the chip formation not only qualitatively but also quantitatively, numerical models have been developed. Lortz [9] presented a surface model of chip formation based on the slip line field theory, where the grain shape was described as a fillet radius of the abrasive grain vertex. This solution was developed by Childs [10], who modeled wedge-shaped grains. In the work of Doman [11], one can already observe two-dimensional analyses of idealized grain shapes. Challen et al. [12], developed models using the theory of the slip line field describing the plastic properties of the deformed material. However, their cutting model was grain-shaped in the form of a conical blade. The authors determined the conditions of the edge geometry and friction on the contact surface at which the material should be machined, and they also provided the relationships for the calculation of stresses in the cut layer.

Torrance [13,14], Badger et al. [15], Williams et al. [16] and Xie et al. [17,18] developed a model of cutting with a single cutting edge in the form of a Vickers pyramid. The models assume that in grinding, the rake surface of the edge is not perpendicular to the cutting direction, and the separation of the processed material takes place as a result of pressing the material laterally in relation to the cutting edge, which results in the formation of not only a chip but also a zone of plastically deformed material on both sides of the scratch. Torrance conducted research on the influence of changes in the orientation of the cutting-edge geometry on the forces and specific energy of cutting, which showed that the lowest energy occurred at angles of attack in the range of 30–60°. He also conducted experimental studies on the chip formation in the cutting process with a Vickers indenter [19].

In order to explain the processes taking place around the vertexes of abrasive grains, tests are carried out using stands that are equipped with image recording systems during the grain operation and with force recorders. For experimental studies, Barge et al. used scratches, where the abrasive grain was placed on a rotating disc, cutting the flat surface of the AlSI4140 steel workpiece [20].

Khellouki et al. [21], in order to determine the relationship between the adhesive friction coefficient and the plastic friction coefficient, performed a single-edge cutting using a scratch test with indenters with a radius of 5 µm and rake angles of 10°, 30° and 45° at a speed of 0.1 mm/s. The authors also conducted tests with a higher speed (60 m/min) and the use of a real abrasive grain, which were carried out with the use of a lathe.

The works of Anderson et al. [22] presented the results of research on scratch profiles and forces, which were recorded on a new proprietary experimental device. On the circumference of a steel wheel with a diameter of 364 mm, a spherical diamond grinding tool with a diameter of 0.508 mm was mounted. The processed material was a transversely mounted ring with a radius of 182 mm, which allowed for the formation of 100 mm long scratches.

In the works of Rasim et al. [23] and Dai et al. [24], the authors analyzed the processes of microcutting with single diamond grains, which were attached to the perimeter of the disc mounted in the spindle of the grinder. The processed sample was placed on a dynamometer, which was mounted on the grinding table. It is worth emphasizing that in the studies by Rasim et al. [23] the microcutting process was recorded with a high-speed camera.

To analyze the chip formation process in grinding, Denken et al. [25] presented a new research method. By using a designed device, they interrupted the chip forming process and proved that chip formation occurred mainly during the contact of large grains with the workpiece.

Modeling material displacements in the microcutting zone is very difficult due to the complexity of the phenomena occurring in the process. The features characterizing not only the contact surface area but also the shape of the contact zone and the conditions of the sample material particles’ displacement depend on the abrasive grain’s depth of cut [23].

The vast majority of research on grinding machining with the use of FEM (Finite Element Method) focuses on the experimental observation and modeling of entire processes related to thermal phenomena, the occurrence of forces on a global scale and energy consumption [26,27,28]. It should therefore be emphasized that all phenomena occurring on the macroscale should be treated as the sum of micro–nano interactions of individual abrasive grains. In order to better understand the grinding process, many authors conduct research in which they analyze the processes of micromachining with single abrasive grains.

One of the first studies using FEM by Doman et al. [29] focused on the use of two-dimensional and three-dimensional models in grinding processes. In the work, the authors discussed the models and presented the approach to assuming boundary conditions and the selection of constitutive material models. The same authors [30] conducted an experimental verification of computer models of the cutting process with a single abrasive grain. They developed a simulation of the cutting process with a single abrasive grain and analyzed typical rubbing and plowing phases. Particular emphasis was placed on the experimental validation of the model by comparing the results of surface properties and forces.

In this article, research on microcutting processes with abrasive grains of a known geometry will be presented. The paper presents a new approach to modeling microcutting processes with single abrasive grains with the use of many complex systems and methods: reconstruction engineering, FEM and microcutting process test stands equipped with a high-speed camera. Understanding the flow of the processed material cut with a single abrasive grain requires the use of all these systems, thanks to which it will be possible to recognize the phenomena occurring in the vicinity of the cutting blades. The aim of the authors was to develop computer models of the microcutting process with a single abrasive grain, in which the separation of the processed material in the form of chips and side ridges (pile-ups) would be validated with the results of experimental tests. The developed models would be the base for further research that would allow one to determine the influence of the geometry of the abrasive grains on the separation phases of the workpiece and the specific energy of the process.

## 2. Materials and Methods

**Figure 1:**
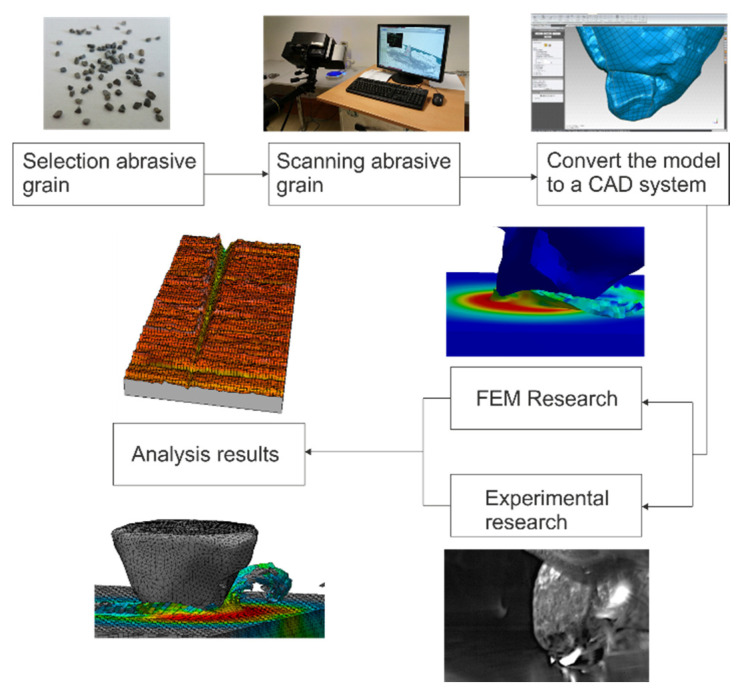
Scheme of the research method of the microcutting process with a single abrasive grain.

In order to analyze the processes of material removal and the phenomena occurring in the zone of abrasive grain contact with the workpiece, simulation studies and experimental tests (to validate the simulation results) were carried out. Reverse engineering tools were used to build the computer model used in the simulation. The stages of creating the model are shown in Figure 1 and discussed in detail in the following sections.

### 2.1. Digital Model of Abrasive Grain

**Figure 2:**
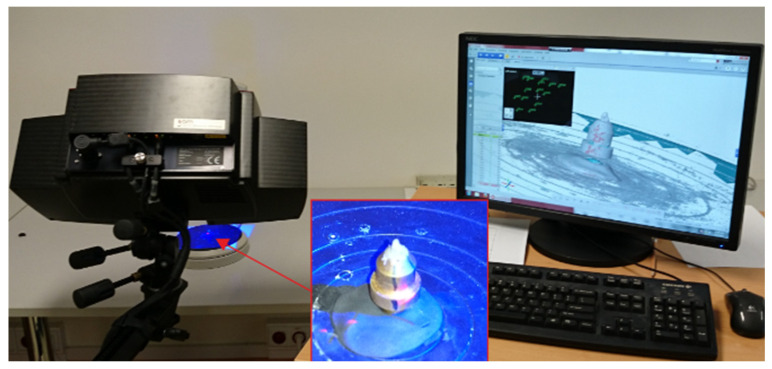
A test stand for scanning abrasive grains.

The geometries of the abrasive grains used in the simulation tests were characterized by a shape consistent with the real grain geometry (grain size 250 µm). The ATOS III SO (manufactured by GOM, Braunschweig, Germany) triangulation scanner, the Geomagic Studio^®^ toolkit (Morrisville, NC, USA) and the Ansys^®^ system (Canonsburg, PA, USA) were used to map the geometrical features selected for the abrasive grain tests, which allowed for the creation of a geometrical model of the abrasive grain. The geometry of the abrasive grain for the tests was prepared by scanning its geometry with the ATOS III SO (Small Object) triangulation scanner (Figure 2). The surface of the abrasive grain was illuminated with a set of blue light lines, creating a grid on the measured surface with a specific density. Patterns in the form of fringe images were recorded by two cameras with a 3296 × 2572 charged coupled device (CCD) matrix. The minimal range between the lines in the patterns used for measuring the abrasive grain was 11.59 µm (single measurement). In order to increase the accuracy of the measurement, 15 images of the measured grain location were made under various angles.

On the basis of optical transformation equations, independent 3D coordinates were determined for each pixel of the recorded image. The geometrical configuration of the detector and distortion parameters was calibrated using photogrammetric methods [31].

In the next stage, the obtained results were processed into the STL format, which enabled importing the geometry to the Ansys^®^ program. For this purpose, the Geomagic Studio^®^ toolkit was used to transform the data from the laser scanner into 3D models. The developed geometric model was imported into the Ansys^®^ system, where it was covered with a finite element mesh.

**Figure 3:**
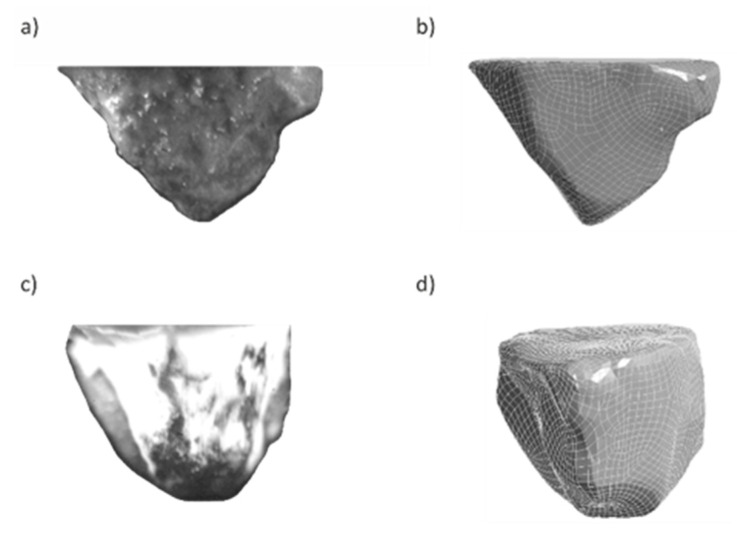
The geometries of the abrasive grains used in the tests: (**a**) grain macrophotography of ZS1, (**b**) geometric model of grain ZS1, (**c**) macrophotography of grain ZS2, (**d**) geometric model of grain ZS2.

In order to verify the correctness of the geometrical models, macrophotographs of the scanned abrasive grains were taken. Macrophotographs and geometrical models of abrasive grains were compared to check the correctness of the scanned geometries. Figure 3 presents the macrophotographs and geometric models of the grains used in the simulation (aluminum oxide abrasive grain ZS1 and ZS2), whose geometrical features coincide with each other, proving the correctness of the tools that were used for digitizing the real objects.

### 2.2. Microcutting Test Stand

**Figure 4:**
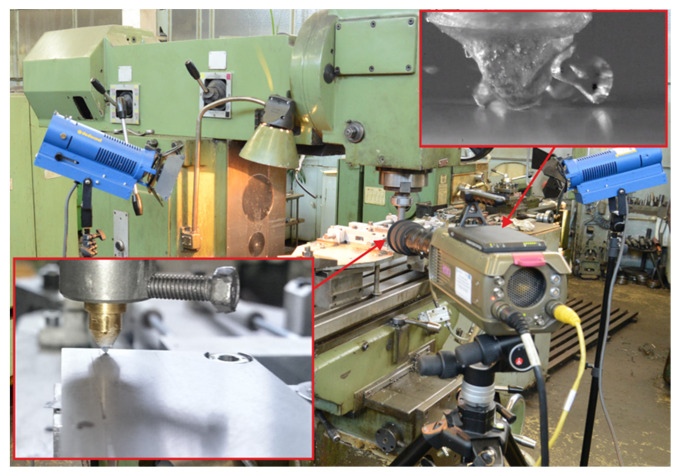
Test stand for scratch tests.

Experimental tests of the microcutting process with a single abrasive grain were carried out on a test stand (Figure 4) equipped with:

- universal milling machine,
- sample feed system with pneumatic drive,
- Phantom v210 high-speed camera with an optical system that enabled ×100 magnification.

The universal milling machine was used to mount the sample feed system, with a pneumatic drive on its table that was responsible for the sample displacement. A handle with the abrasive grain was mounted in the spindle of the milling machine. The spindle was previously blocked against rotation. The pneumatic linear drive was supplied directly from an expansion tank with a volume of 200 L and pressure of 9 bar. Thanks to this, a constant pressure was ensured, which allowed the tested samples to be moved at a speed of vs = 10 m/s. The control system of the milling machine enabled the precise positioning of the abrasive grain in relation to the sample on which the scratches were made.

The Ti-6Al-4V titanium alloy sample was tilted to an angle α 0.02°, and its length was 90 mm. The slight inclination allowed one to obtain information on the influence of the penetration of the abrasive grain (in the range of 0 to 25 μm) on the form of the chip and ridges. The inclination of the sample also allowed one to avoid breaking the abrasive grain in the initial phase of contact with the workpiece.

An integrated high-speed image recording system was used to study the material removal process in the microcutting process with a single abrasive grain. The system included the Phantom v12.1 high-speed camera, whose acquisition parameters allowed one to record up to 1 million frames per second, and the maximum filming resolution was 1280 × 800 pixels at a sensitivity of 6400 ISO/ASA with a minimum exposure time of 300 ns. In the experimental studies, the microcutting process was recorded at a resolution of 1024 × 512 pixels, with a write speed of 11,854 fps and an exposure time of 41 μs. Spot lighting with a brightness of 4.6 million lux and an optical set enabling magnification of up to 100× were used.

The cutting path profile was measured with a Talysurf^®^ CLI 2000 (manufactured by Taylor Hobson, Leicester, UK) spatial profilometer. The measurements were performed with the use of the CLA-300 non-contact laser sensor, which enabled a measurement of inequality in the range of 646 pm–8.7 mm. Microtopographic measurements were made in 10,000 passes with a 0.5 μm step. In one pass, about 1000 points were recorded every 1 μm at a table speed v = 500 μm/s. The measurement data was analyzed in the TalyMap^®^ program, and the results of that work are presented later in the article.

In order to correctly assess and observe the chip formation, the authors wanted the cutting path to be as long as possible in the experiment. The scratch length for microcutting with the ZS1 grain was 77 mm, and for the ZS2 grain it was 79 mm. In the processes of grinding with grinding wheels, the length of the machining marks depends on the grinding depth and is in the range of 0.2 to 5 mm. On the other hand, in the smoothing processes with abrasive foils, the contact length of the grain with the processed material can even be 100 mm. Recorded data from experimental and simulation tests in the form of scratch cross-sections will be compared with each other later in the work. Due to the long scratches, measurements of the bottom cross-sections and pile-ups were carried out in the area where the blade recess was 10 μm, 15 μm and 20 μm.

### 2.3. FEM Model of the Cutting Process

The computer model was developed using the Ansys^®^ Autodyna system, according to the following stages:

- Based on the scanned abrasive grain geometry, a geometric model of the tool-workpiece system was created.
- The calculation method and the discretization scale were defined.
- The material model (Ti-6Al-4V) of the workpiece was defined.
- The type of contact between the tool and the processed material was selected.
- The boundary conditions were defined.

The purpose of the simulation tests was to analyze the formation of ridges and chips in the process of microcutting with single abrasive grains. The development of computer models that fully describe the material removal process is extremely difficult due to the complexity of the phenomena occurring in the microcutting zone. Therefore, the numerical analyzes were simplified. The physical properties of the abrasive grains were ignored and modeled as perfectly rigid bodies. The workpiece was defined as homogeneous throughout its volume.

The difference between the conditions of the experiment and the simulation also concerned the range of displacements of the abrasive grain along the microcutting path. Due to the limitations of the computing power of the workstation, the length of the scratch created during the simulation of the microcutting process was reduced to 15 mm, and a larger angle of workpiece inclination α was adopted in order to obtain a cutting depth comparable to that in the experimental tests.

The sample material was modeled as viscoplastic (the number of finite elements was 201,345). The object was discretized with eight nodal Solid164 elements. For the abrasive grain ZS1 microcutting process, the number of finite elements was 241,825, and for the abrasive grain ZS2 it was 244,633. The difference in the number of finite elements was caused by the different abrasive grain geometries. The number of finite elements in the workpiece was the same. Translational and rotational degrees of freedom were removed for the nodes at the base of the sample. The initial speed of the abrasive grain speed was set to 10 m/s.

### 2.4. Constitutive Equation

The applied constitutive material model relates to the flow of stresses, strains, strain rates and temperature distribution in the tested sample, and its parameters are presented in Table 1. Johnson–Cook equations [32,33,34,35,36,37,38,39] are commonly used to model materials that are exposed to deformation in a wide range of strain rates and temperatures. The general form of the Johnson–Cook equation is [38,40]:

(1) $$\sigma =\left(A+B{\left(\varepsilon_{p}\right)}^{n}\right)\left(1+C\;\times \;ln\frac{{\dot{\varepsilon }}_{p}}{{\dot{\varepsilon }}_{0}}\right)\left(1-{\left(\frac{T-T_{amb}}{T_{melt}-T_{amb}}\right)}^{m}\right)$$

where *A*—initial, static yield strength; *B*—parameter of plastic strength; *ε_p_*—effective plastic strain; *n*—exponent of plastic deformation strength; *C*—material parameter specifying the impact of the intensity of the plastic speed deformation;$\;{\bar{\varepsilon }}_{p}$, ${\dot{\varepsilon }}_{0}$—effective plastic and reference strain rates; *T*, *T_amb_*, *T_melt_*—current, ambient and melting temperatures; and *m*—exponent of thermal plasticity.

The simulation was performed using the Ansys^®^ Autodyna system for the spatial deformation state. Solving the posed problem of determining the stresses and displacements of the workpiece material, the explicit method was used. In this method, the equation describing the movement of the object can be written as:

(2) $$M\ddot{r}\left(\tau \right)+C\dot{r}\left(\tau \right)+Kr\left(\tau \right)=R\left(\tau \right)$$

where: ***M***, ***C*** and ***K*** are the respective constants in time matrices of the mass, damping and system stiffness. ***R*** expresses the internal load vector, and $\ddot{r}$, $\dot{r}$ and r are, respectively, the vectors of displacement, rate and acceleration of the nodes of the system.

## 3. Results and Discussion

**Figure 5:**
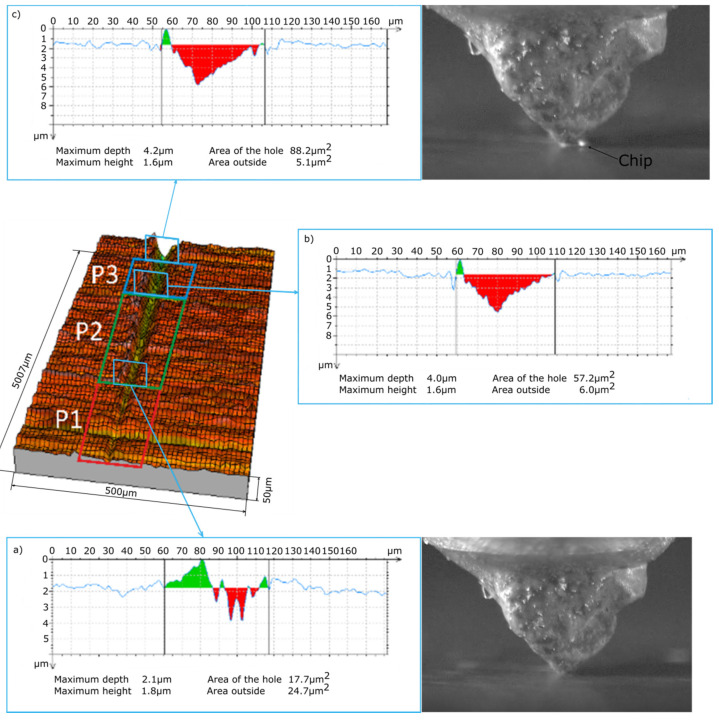
View of the initial stage of the scratch formed by the ZS1 abrasive grain and cross-sections of the scratch at the end of the stages: (**a**) P1, (**b**) P2 and (**c**) P3.

**Figure 6:**
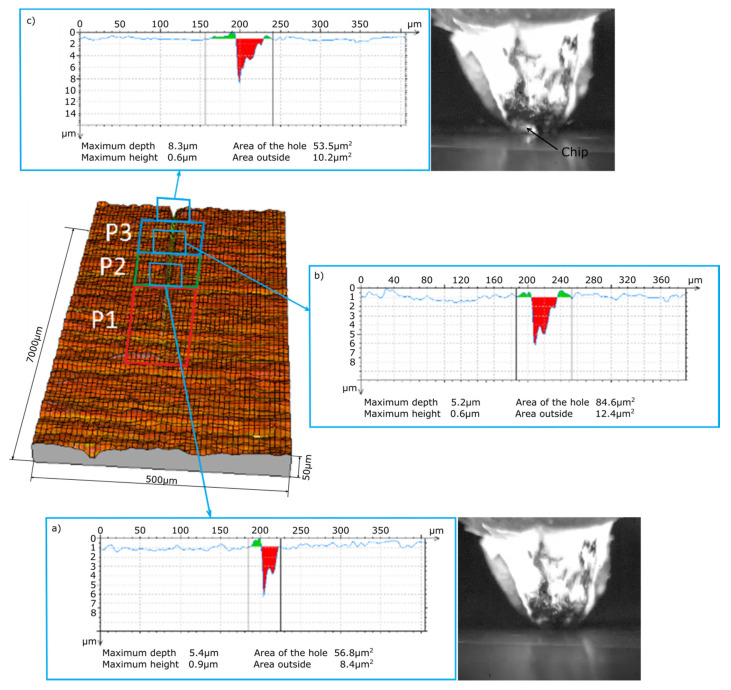
View of the initial stage of the scratch formed by the ZS2 abrasive grain and cross-sections of the scratch at the end of the stages: (**a**) P1, (**b**) P2 and (**c**) P3.

In the processes of cutting with the single abrasive grains ZS1 and ZS2 recorded with a high-speed camera, three stages can be observed that are typical for the plowing process (P1), in which the processed material has a large predominance of plastic deformation, the transition area (P2), where the grain starts cutting, and the material separation stage in the form of a chip (P3). Figure 5 and Figure 6 show stages P1, P2 and P3 for microcutting with the single abrasive grains ZS1 and ZS2. By observing the scratches and film sequences, it can be observed that for two different geometries, the following stages occur at different distances from the first contact with the material. The length of the P1 zone (before chip formation) for the ZS1 and ZS2 grains is, respectively, 1764 µm and 2413 µm. It can therefore be concluded that the grain size alone does not determine the path of microcutting along which the separation of the material will occur in the form of a chip. Thus, the chip separation process is influenced by the geometric features of the single abrasive grain and the grain orientation in the cutting direction.

**Figure 7:**
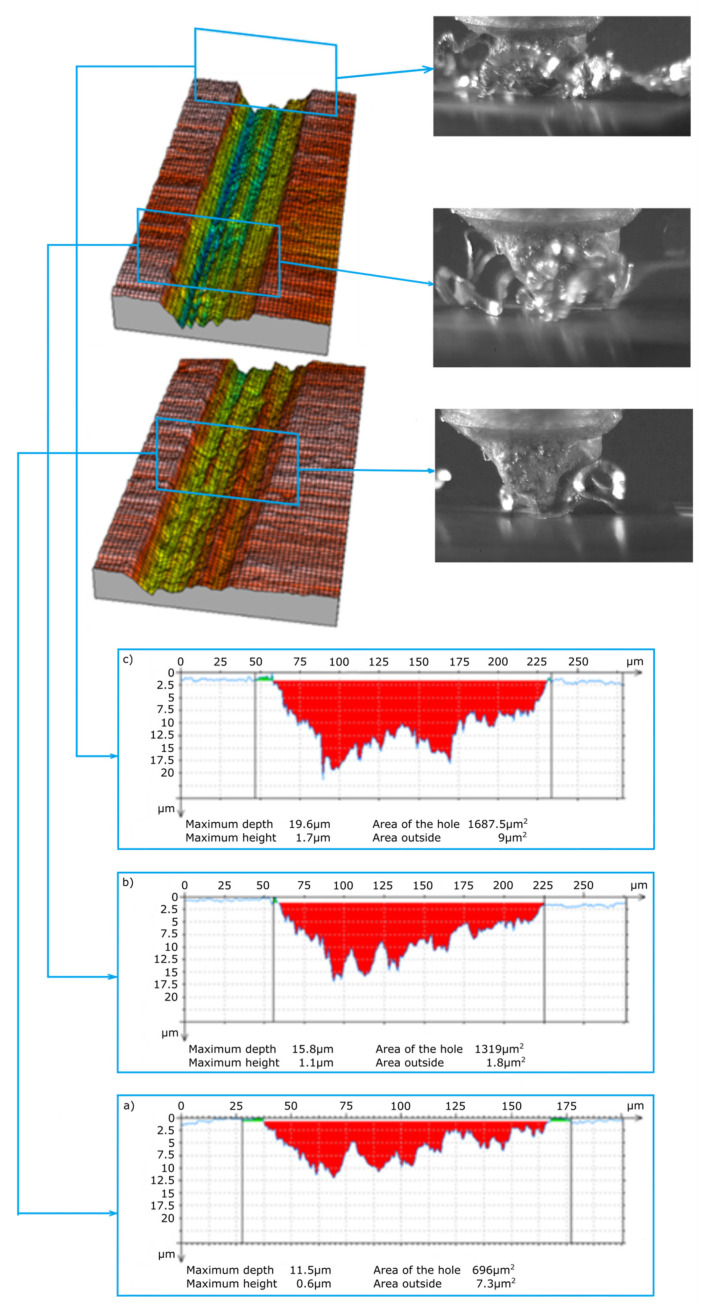
View of the scratch formed by the ZS1 abrasive grain and cross-sections of the scratch for depths of cut of (**a**) 10 μm, (**b**) 15 μm and (**c**) 20 μm.

**Figure 8:**
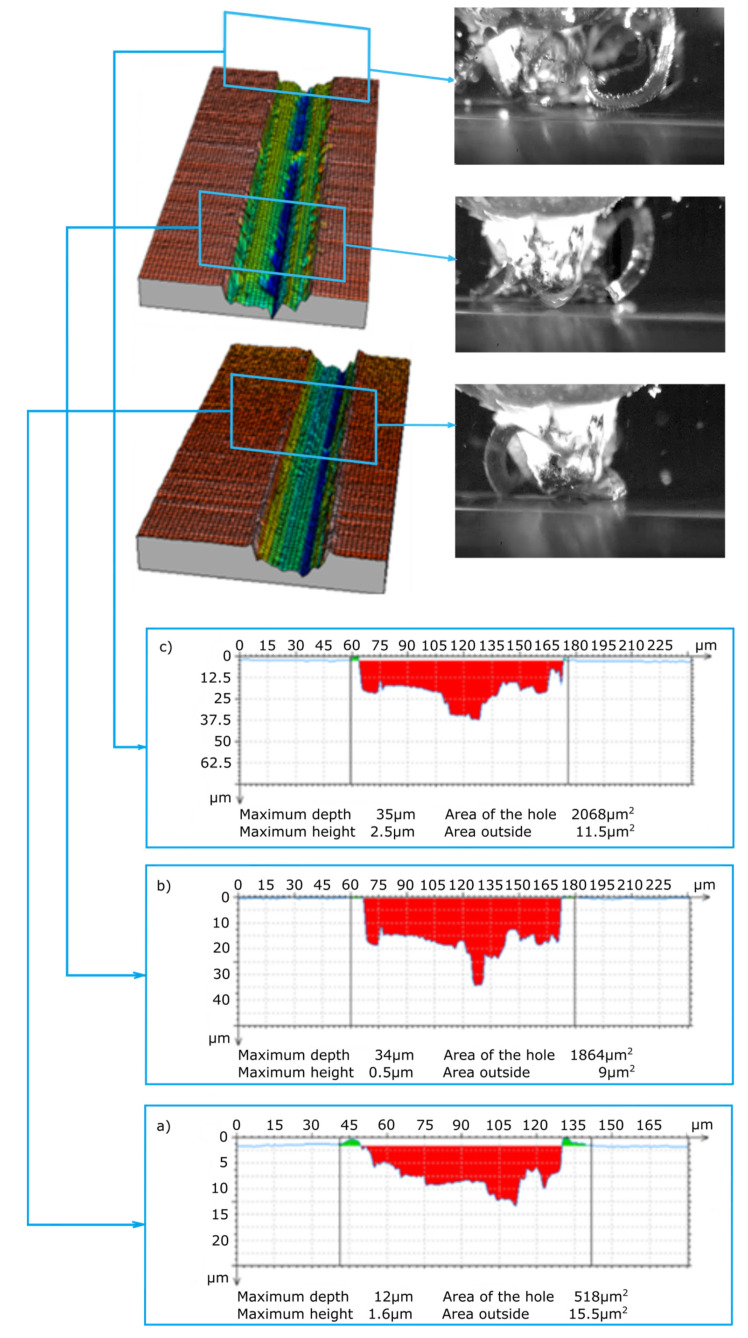
View of the scratch formed by the ZS2 abrasive grain and cross-sections of the scratch for depths of cut: (**a**) 10 μm, (**b**) 15 μm and (**c**) 20 μm.

The abrasive grains selected for testing (Figure 3) were oriented in such a way that the wide grain edge was perpendicular to the cutting direction. This is an advantageous arrangement due to the geometry and orientation of the grain causing the continuous separation of the material in the form of a wide ribbon chip. This is confirmed by the frames shown in Figure 7 and Figure 8 from the images recorded with a speed camera with a magnification of 90×. The long and wide chips shown in the photos are characteristic of a continuous chip flow in front of the wide rake face of the grain. In addition, the ZS1 grain has the geometric features of an asymmetrical wedge, which, for a larger depth of cut, causes a lateral chip deposition. On the other hand, the geometrical features of the grain have concavities favorable for the separation process, which facilitate the flow of the processed material in the form of a ribbon chip. An unfavorable phenomenon that can be observed in the images from the film recorded with a high-speed camera is the wrapping of ribbon chips around the grain. This phenomenon could be observed after the grains ZS1 and ZS2 traveled a 30-mm distance (the depth of cut was about 10 μm). Thus, it can be assumed that grains with a preferred geometry, which separates the material in the form of a ribbon chip, can significantly accelerate the adhesion of the active surface of the grinding wheels or the abrasive foil.

The main purpose of examining the traces of machining after micromachining with ZS1 and ZS2 grains of Ti-6Al-4V titanium alloy was to check the scratch profile and the size of the pile-ups, the results of which are presented in Figure 5, Figure 6, Figure 7 and Figure 8. As already mentioned, the results of the cross-sections of the scratch bottom are presented for depths of cut of 10 μm, 15 μm and 20 μm.

With a small depth of cut of the ZS1 and ZS2 grains (Figure 5 and Figure 6) into the workpiece, for which the separation of the material in the form of a chip had not yet occurred, high plastic deformations in the P1 zone resulted in the formation of side outflows of material of a considerable size in relation to the cross-sectional area scratches. The plastically deformed workpiece was moved in front of the abrasive grain and unevenly deposited in the form of side pile-ups on the sides of the cutting trace. At the end of the P1 zone, cross-sectional to the cutting direction, the side-discharge area for grain ZS1 is 24.7 μm^2^, and for grain ZS2 it is 8.4 μm^2^. A significantly larger area of side outflows for the ZS1 grain results from the shape of the abrasive grain tip. This tip has the shape of a cone, which promotes the movement of the processed material to the sides of the scratch. The ZS2 grain has a flat tip with a wide cutting edge, which makes it difficult to move the material to the sides of the scratch. Significant changes in the cross-section size of the analyzed abrasive grains in the initial stage of machining are clearly indicated by the cross-sectional area of the scratch, amounting to 17.7 μm^2^ for the ZS1 abrasive grain and 56.8 μm^2^ for the ZS2 abrasive grain.

The cross-sections of the cutting layer obtained from the blade penetrating into the processed material at 10 μm, 15 μm and 20 μm (Figure 7 and Figure 8) indicate different sizes of side pile-ups for the ZS1 and ZS2 grains. The shape of the ZS1 grain, having a flat and wide cutting surface, makes it difficult to move the workpiece sideways and favors chip formation (which confirms the results of previous research on grinding with abrasive aggregates [40]). The average surface area of the side pile-ups in relation to the scratch surface is about 0.5% for the ZS1 grain, while for the ZS2 grain it is about 0.8%. The cross-sections of the scratches shown in Figure 7 and Figure 8 are characterized by irregular curves, which indicate the chipping of abrasive grain vertexes. A clear change in the cross-section of the scratch bottom after the ZS1 grain has passed through (Figure 7) is particularly visible. As the grain goes deeper and deeper into the workpiece, the irregularity and variability of the cross profile of the scratch bottom are visible.

**Figure 9:**
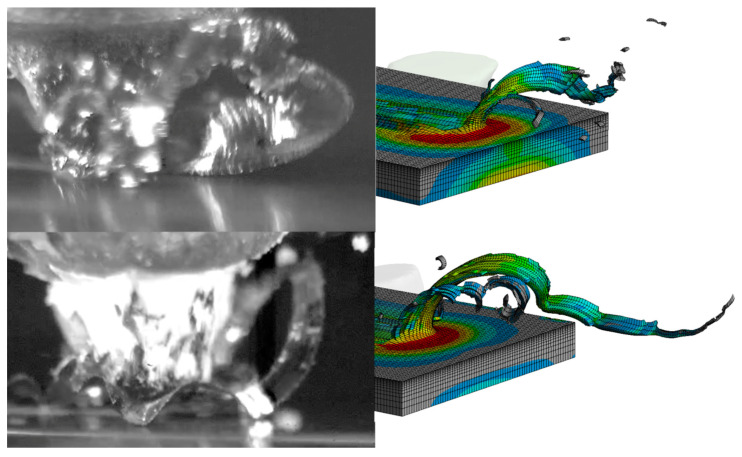
View of the chip formation in the cutting process with the (**a**) ZS1 grain and (**b**) ZS2 grain.

When analyzing the results of the simulation tests, attention should be paid to the great similarity in the method of chip formation. A comparison of images recorded with a high-speed camera and the results of the simulation tests is shown in Figure 9. In the simulation tests, the orientation of the grain in relation to the cutting direction was consistent with the grain orientation in the experimental tests, which contributed to the convergence of the results of the chip and pile-up formation process. As already mentioned in Section 2, the shorter cutting path in the simulation tests is due to limitations in the computing power, which results in an inability to compare the chip shaping mechanism with the experimental tests for the same cutting path. However, it is possible to observe the similarity in the very method of forming a ribbon chip and to analyze the cutting efficiency for a specific penetration of the blade into the workpiece. The geometric features and orientation of the ZS1 grain cause the formation of wide ribbon chips with a tendency to twist in a helix. On the other hand, the features of the ZS2 grain and its orientation result in the formation of ribbon chips, resembling a shape that is typical for the orthogonal turning process.

**Figure 10:**
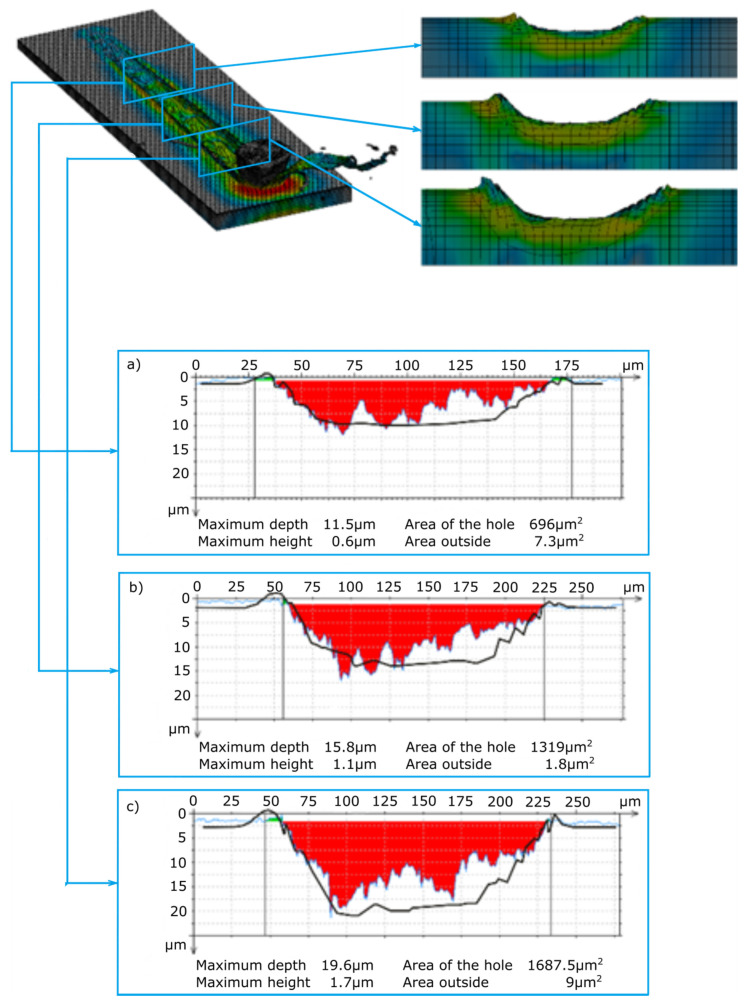
Results of the profiles of the scratch formed in the process of microcutting with the ZS1 grain (simulation tests); (**a**) validation of the scratch profiles in the simulation tests for a depth of cut of 10 μm, (**b**) validation of the scratch profiles in the simulation tests for a depth of cut of 15 μm, and (**c**) validation of the scratch profiles in the simulation tests for a depth of cut of 20 μm.

**Figure 11:**
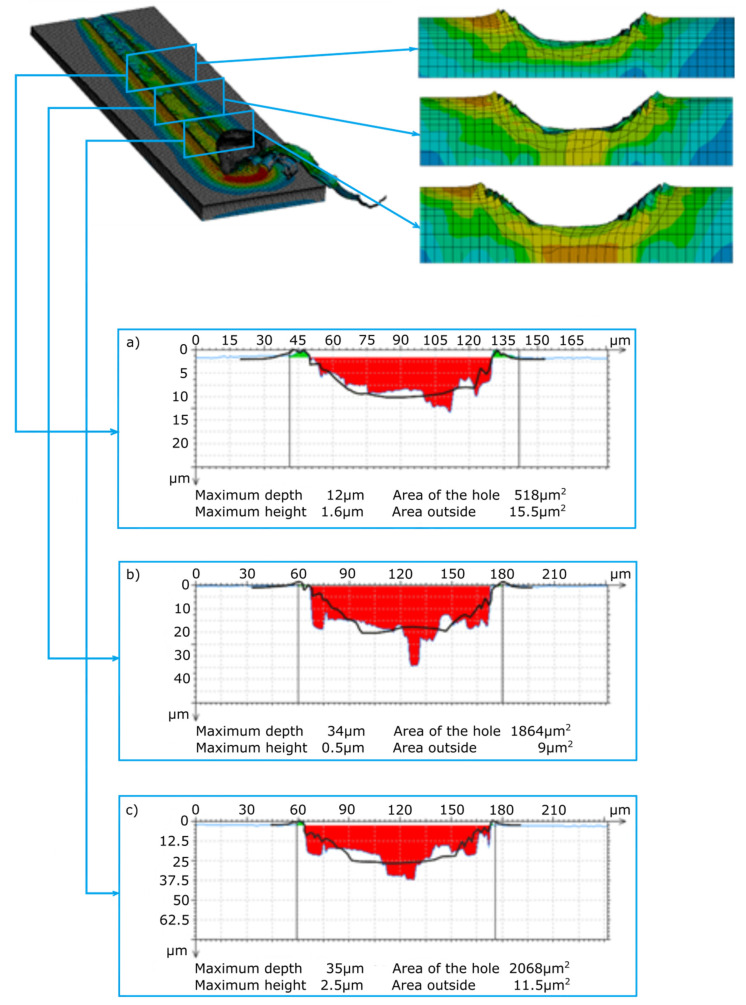
The results of the profiles of the scratch formed in the process of microcutting with the ZS2 grain (simulation tests); (**a**) validation of the scratch profiles in the simulation tests for a depth of cut of 10 μm, (**b**) validation of the scratch profiles in the simulation tests for a depth of cut of 15 μm, and (**c**) validation of the scratch profiles in the simulation tests for a depth of cut of 20 μm.

Figure 10 and Figure 11 present the results of the measurement of scratches from simulation tests after microcutting with the ZS1 and ZS2 blade of the Ti-6Al-4V titanium alloy. The scratch profiles from the simulation tests were transferred to the scratch profiles from the experimental tests, which are marked with a bold black line (Figure 10a–c and Figure 11a–c). The scratch profiles obtained in the course of the experimental and simulation tests are similar in terms of the width and depth of the cut. The greatest convergence of the results of the scratch cross-sections measured in the simulation and experimental tests can be observed for the cutting process with the ZS2 grain (Figure 11a), where with a depth of 10 μm, scratch widths are the same and amount to 84 μm. A large convergence of results was observed for the width and height of the side pile-ups. The greatest convergence was observed for the 10 μm depth of cut, where the width and height of the left pile-ups were 3 μm and 1.5 μm for the experimental tests, 3.5 μm and 1.5 μm for the simulation tests, and the same for the right outflow for the experimental and simulation tests, amounting to 3.5 μm and 1.5.

## 4. Conclusions

The geometrical features of the abrasive grains and their orientation in the cutting direction significantly affect the material removal process, which in turn affects the topography of the machined surfaces. During the microcutting processes, the active grain vertexes wear out and break, which affects the profile of the machining traces and the mechanisms of material separation. The article presented a new approach to simulation studies of microcutting processes, in which methods used in reconstruction engineering were applied, thanks to which it was possible to conduct a more detailed research on the mechanisms of material separation in grinding processes. Based on the results of the experimental and simulation studies presented in the article, the following detailed conclusions were drawn:

- The geometrical features of the abrasive grains and their orientation towards the microcutting direction influence the length and starting point of each of the three typical grinding stages: rubbing, plowing and chip formation.
- Orienting the abrasive grain with the wide edge of the grain perpendicular to the direction of the cut is a preferred setting. This has the effect of continuously separating the material in the form of a wide ribbon chip.
- The use of a precise triangulation optical scanner to evaluate the geometric features of the abrasive grains and the possibility of processing the grain into a digital model enables the digital import, consistent with the real geometry, to the system using the FEM. Thanks to this, it is possible to get to know the processes/mechanisms of chip, pile-up and scratch formation.
- The analysis of the convergence of the simulation and experimental studies showed (despite the simplifications introduced in the FEM simulation resulting from the computing power) that the results of the scratch width and the size of the outflows largely coincided with each other, especially for a grain depth of 10 μm. Therefore, it can be concluded that the methods, models and constitutive equations used in the experimental and simulation studies are correct and that their application allows one to learn about the processes occurring in the vicinity of abrasive grain vertexes.

Based on the analysis of the convergence of the simulation tests with the experiment, it can be concluded that the material model used in the simulation tests and the constitutive equations are correct and that their application allows one to learn about the processes occurring in the vicinity of abrasive grain vertexes. The authors are already working to determine the influence of the variability of geometrical features depending on the grain depth in the processed material. It is worth emphasizing here that, depending on the depth of cut, the geometrical features of grains are different and significantly affect the process of pile-ups and chip formation. This study is the basis for further research related to the understanding of the phenomena occurring in the vicinity of the abrasive grain vertexes, which, thanks to the new approach, opens up new possibilities in the assessment of the mechanisms of ridge and chip formation.

## Figures and Tables

**Table 1:** Parameters of the model used in the simulation [30].

Material	A, MPa	B, MPa	C	n	m
Ti-6Al-4V	820	220	0.26	0.01	1.01
